# Umbilical Endometriosis With Appendiceal Involvement: A Case Report

**DOI:** 10.7759/cureus.87473

**Published:** 2025-07-07

**Authors:** Varna Jammula, Kevin Johnson, Matthew Grolle

**Affiliations:** 1 College of Osteopathic Medicine, Touro University Nevada, Henderson, USA; 2 Obstetrics and Gynecology, Women's Health Associates of Southern Nevada, Las Vegas, USA

**Keywords:** appendiceal endometriosis, dysmenorrhea, multifocal endometriosis, umbilical endometriosis, umbilical mass

## Abstract

Endometriosis is a common disease that affects women worldwide. While it is most commonly located in the pelvis, lesions may occur in other areas of the body. Concurrent umbilical and appendiceal endometriosis is rare. We present a case of umbilical endometriosis with appendiceal involvement in a 29-year-old female. The patient presented with a periumbilical mass that bled during her menstrual cycle. She also reported lower quadrant pelvic pain, dysmenorrhea, and dyspareunia. Imaging revealed an umbilical hernia and a lesion abutting the suspensory ligament of the right ovary. Cancer antigen-125 (CA-125) levels were elevated. Diagnostic laparoscopy revealed stage IV endometriosis with severe adhesions to the uterus, posterior cul-de-sac, descending and sigmoid colon, and bladder. An incidental appendiceal mass was noted, resulting in an appendectomy. The periumbilical mass was removed. Pathology revealed umbilical and appendiceal endometriosis. There were no complications in the postoperative period. The diverse manifestations of endometriosis emphasize the importance of considering extrapelvic involvement, especially in patients with atypical dermatological symptoms. Early recognition and subsequent laparoscopy for both the diagnosis and treatment of endometriosis are essential for successful patient management.

## Introduction

Endometriosis is a common inflammatory disease characterized by the presence of lesional endometrial tissue outside of the uterus. These lesions are known to cause cyclical pelvic pain, infertility, and may lead to heavy or prolonged menstrual bleeding [1]. The most common locations for endometrial lesions are intrapelvic sites such as the ovaries, the broad ligament, the anterior and posterior cul-de-sac, and the uterosacral ligament; however, they are by no means limited to the pelvis [1]. While the exact etiology of endometriosis is unclear, several theories have been proposed. Sampson's retrograde theory, the coelomic metaplasia theory, and the lymphatic and vascular infiltration theory are some of the most widely discussed [2]. Sampson's theory, considered one of the most plausible, posits that menstrual tissues can flow retrograde through the fallopian tubes and implant within the peritoneal cavity, where they can grow, infiltrate local tissues, and lead to chronic inflammation. The gold standard for diagnosis remains laparoscopy with histological confirmation. Unfortunately, there are no laboratory tests that are specific for endometriosis, so non-invasive diagnosis depends heavily on diligent history-taking and physical exam findings, which, by themselves, cannot always rule out the disease. Treatment can be divided into two primary categories. Medical treatment with hormonal and non-hormonal agents to reduce symptoms and spare fertility, and surgical treatment to remove the extra-uterine endometrial tissue [3]. It is thought that endometriosis is present globally in around 10% [4,5] of women of reproductive age. While lesions are generally confined to the pelvis, they can occur anywhere in the body. Appendiceal and umbilical endometriosis are each rare occurrences, found in approximately 2.8% [6] and 0.5%-1% [7] of all endometriosis cases, respectively. Umbilical endometriosis presents with an umbilical mass, and cyclical menstrual-related pain, swelling, and bleeding from the umbilicus. Appendiceal endometriosis can confusingly mimic the symptoms of appendicitis, albeit in a cyclical manner; sudden right-sided lower abdominal pain, nausea, vomiting, and fever. Having either of these presentations is an uncommon occurrence, and having them appear together is exceedingly rare, with only two cases documented in the last 10 years [8,9]. The unusual presentation and rarity of multi-loci non-pelvic endometriosis present a diagnostic and surgical challenge requiring prompt diagnosis and a multidisciplinary approach. We present a case of umbilical endometriosis with appendiceal involvement in a 29-year-old female.

## Case presentation

A 29-year-old female presented with an abnormal umbilical growth, dysmenorrhea, and bilateral lower quadrant pelvic pain. She described the pain as cramping in nature and worsens during intercourse. Her menstrual cycles were regular, with heavy bleeding. The umbilical mass was noted in 2023 and tripled in size in one year. The patient complained of bleeding from the mass during her menstrual cycle. She had tried oral contraceptive pills (both progestin-only and combined hormonal), progesterone injections, and nonsteroidal anti-inflammatory drugs (NSAIDs), all of which provided minimal relief. She had one prior vaginal delivery and a surgical history of pleurodesis for a right spontaneous hydropneumothorax. On physical exam, the abdomen was soft, non-tender, and non-distended with a large pigmented umbilical skin lesion. Tumor marker laboratory showed an elevated cancer antigen-125 (CA-125) level and normal carbohydrate antigen 19-9 (CA 19-9) and carcinoembryonic antigen (CEA) levels (Table 1). A transvaginal and abdominal ultrasound revealed a possible polyp (measuring 1.2 cm x 0.4 cm x 0.9 cm), left ovarian complex cyst (measuring 3.9 cm x 2.9 cm x 3.7 cm), and umbilical mass. Three few weeks later, abdominal and pelvic MRI revealed large-volume T1 hyperintense ascites, an umbilical hernia, a T2 hypointense lesion abutting the suspensory ligament of the right ovary (measuring 1.7 mm), and bilateral ovarian follicles and cysts. Due to concerns of malignancy and severity of the symptoms, surgical intervention was deemed to be the best next step in management. 

**Table 1:** Routine laboratory tests and tumor markers. * Non-smoker range. Hct, hematocrit; Hgb, hemoglobin; RBC, red blood cells; Plt, platelets; CA-125, cancer antigen-125; CA 19-9, carbohydrate antigen 19-9; CEA, carcinoembryonic antigen

Laboratory tests	Values	Reference range
Hct	31.70%	35.00-60%
Hgb	10.20 g/dL	11.00-18.00 g/dL
RBC	3.72 x 10^3^/mL	4.00-6.00 x 10^3^/mL
Plt	451 x 10^3^/mL	150-450 x 10^3^/mL
CA-125	55 U/mL	<35 U/mL
CA 19-9	<3.0 U/mL	<34 U/mL
CEA	<2.0 mg/mL	<2.5 mg/mL*

**Figure 1:**
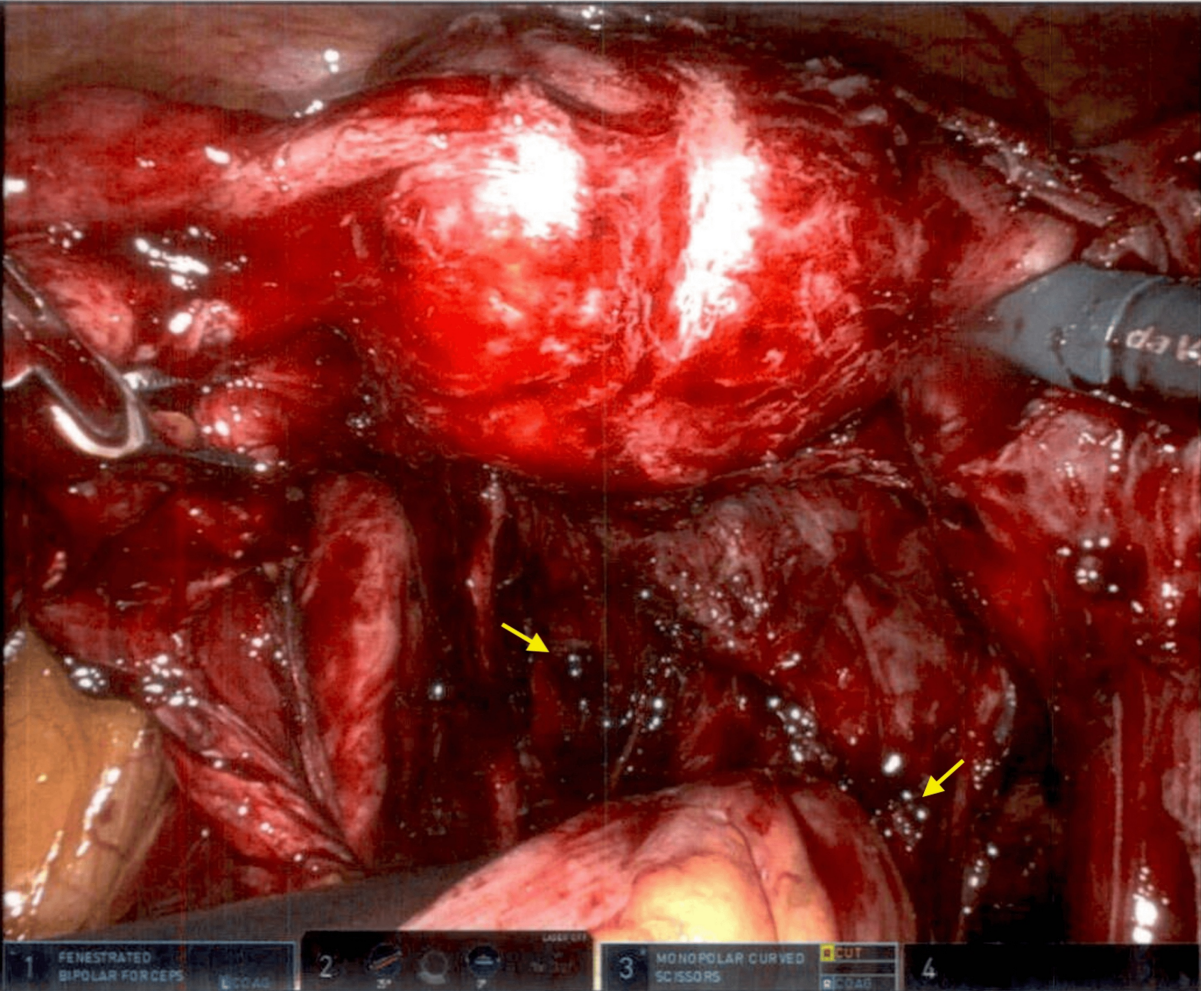
Stage IV endometriosis. Endometrial lesions (yellow arrows) were noted intraoperatively when removing adhesions and restoring the posterior cul-de-sac.

**Figure 2:**
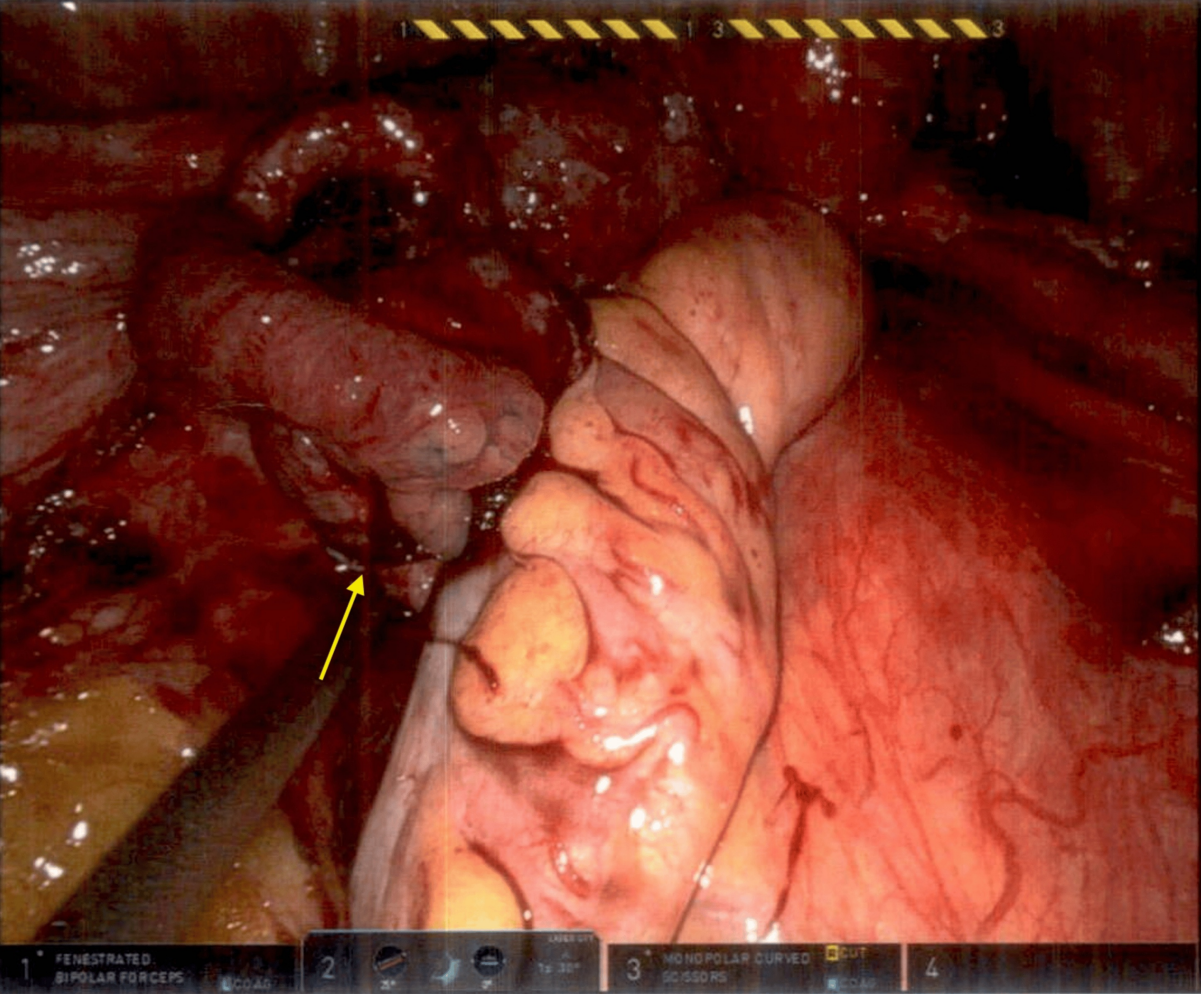
Appendiceal mass with abnormal anatomy. A mass (yellow arrow) with abnormal anatomy was noted intraoperatively involving the appendix.

**Figure 3:**
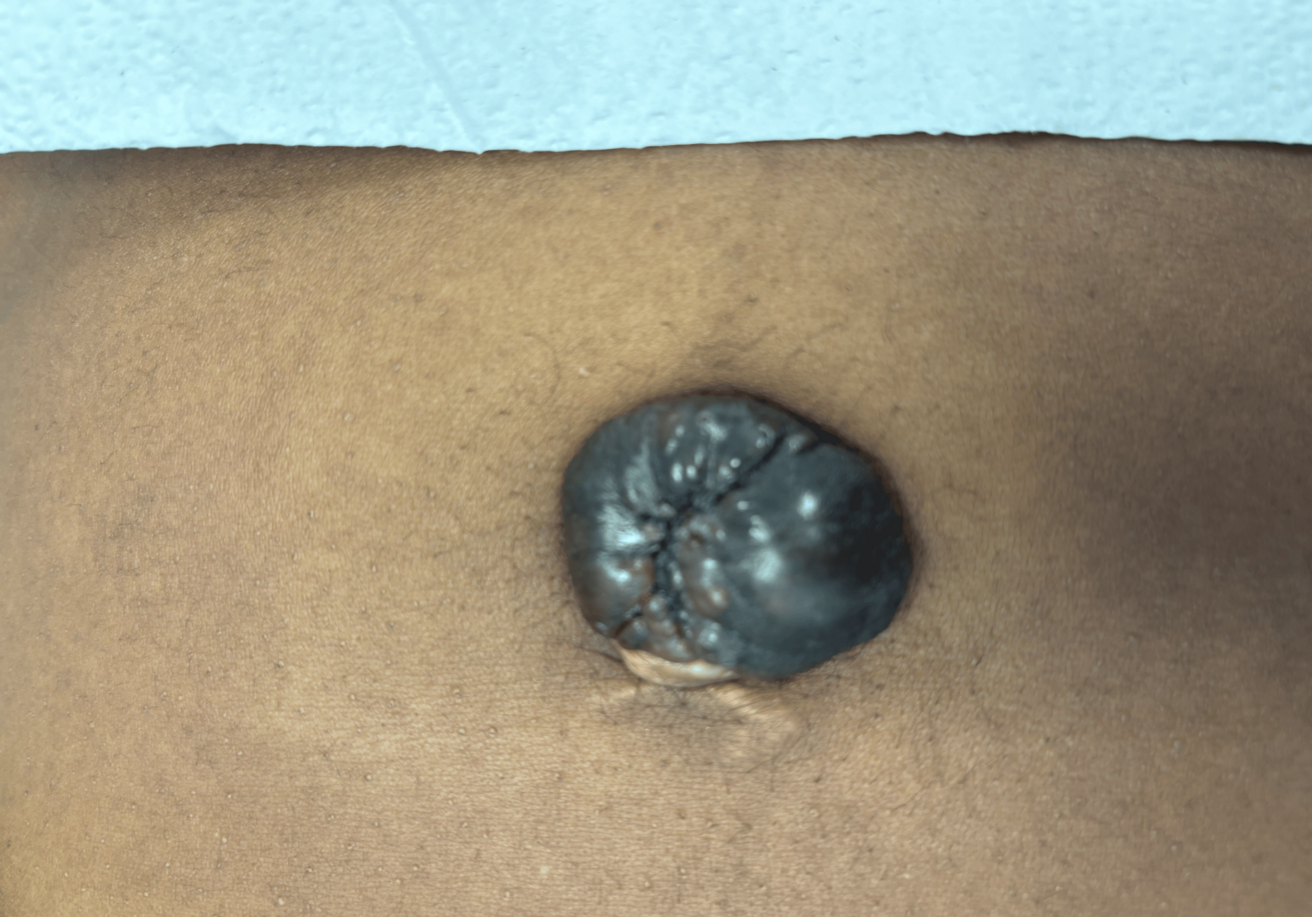
Umbilical mass.

A robotic-assisted diagnostic laparoscopy was performed with resection of the periumbilical mass. Stage IV endometriosis was noted with severe adhesions to the uterus, posterior cul-de-sac, descending and sigmoid colon, and bladder (Figure 1). Serosanguineous fluid was removed from the abdomen, most likely due to a ruptured endometrioma. The fenestrated bipolar and monopolar shears were utilized to carefully remove adhesions and dissect retroperitoneally to localize the left and right ureters near the infundibulopelvic ligament. The sigmoid colon was carefully dissected off the uterus, and restoration of the posterior cul-de-sac was then performed. The anterior bladder was further dissected off the uterus, and fulgurations were performed on the endometrial implants. Bilateral hydrosalpinx was noted, and a chromopertubation was performed with visualization of left tube patency. An incidental appendiceal mass was noted with abnormal anatomy, prompting a general surgery consult and subsequent appendectomy with lymph node dissection (Figure 2). A 3 x 3 cm umbilical mass was removed (Figure 3). On pathological examination, the periumbilical mass was described as a wrinkled polypoid polypoid-appearing portion of skin that is focally finely granular. Pathology revealed appendiceal and periumbilical masses consistent with endometriosis and a benign periappendiceal lymph node. The patient was discharged home on the same day with no complications. She was seen in the clinic three weeks later with well-healing incision sites and no complaints.

## Discussion

Endometriosis is a chronic disease that is often debilitating and presents with significant diagnostic and therapeutic challenges. Without treatment, fertility can be compromised, with nearly 50% of women with endometriosis being infertile [10]. Additionally, due to the chronic nature of endometriosis-associated pain, it is associated with an increased risk of depression and anxiety [11]. Although endometriosis is common, its location can vary throughout the pelvic cavity, most commonly in the ovaries, uterosacral ligaments, and posterior cul-de-sac [12]. Several theories to explain ectopic implantation of endometrial tissue involve the migration of endometrial cells through the peritoneal cavity, lymphatic system, vasculature, or embryonic remnants [7]. The presence of extrapelvic endometriosis is rare; only 0.5%-1% [7] and 2.8% [6] of all cases have been reported as umbilical endometriosis and appendiceal endometriosis, respectively. This case highlights an unusual presentation of synchronous umbilical and appendiceal endometriosis.

While the patient’s clinical presentation may aid in the diagnosis of endometriosis, the gold standard for diagnosis is still laparoscopy. Umbilical endometriosis may often be misdiagnosed as a dermatological condition such as a keloid, dermatofibroma, or Sister Mary Joseph nodule [13,14]. Appendiceal endometriosis may mimic the signs of acute appendicitis due to the characteristic right lower quadrant pain, nausea, and vomiting [15]. Therefore, histopathological examination is crucial in the definitive diagnosis of both umbilical and appendiceal endometriosis. Imaging is helpful in the evaluation of suspected endometriosis to provide additional detail on the extent of the disease before laparoscopy. In this case, MRI and transvaginal ultrasound revealed an umbilical mass and endometrioma. Additionally, this patient had an elevated CA-125, a marker frequently associated with both endometriosis and ovarian malignancy [16]. However, appendiceal endometriosis was not an expected finding since the patient did not report right lower quadrant pain, and imaging did not detect an appendiceal mass. Laparoscopy and histopathological examination provided the definitive diagnosis of endometriosis and its locations in this patient. 

This case is significant because it highlights the rare synchronous occurrence of umbilical and appendiceal endometriosis. Further, we stress the need for a multidisciplinary approach, including gynecologists, general surgeons, and radiologists, in evaluating and managing complex cases of endometriosis with extrapelvic foci. Given that both conditions are individually uncommon and their coexistence is exceptionally rare, this case underscores the importance of heightened clinical awareness and the consideration of extrapelvic endometriosis in patients with appropriate risk factors, history, and cyclical pelvic and abdominal pain. 

## Conclusions

Endometriosis remains a complex and often underdiagnosed condition, particularly when presenting outside the pelvis. This case of multifocal umbilical and appendiceal endometriosis demonstrates the diverse manifestations of the disease and the importance of considering extrapelvic involvement in patients with atypical dermatological symptoms. The successful management of this patient underscores the role of laparoscopic intervention in both diagnosis and treatment. Moving forward, increased awareness and further studies in extrapelvic endometriosis may help improve early recognition and lead to more tailored treatment approaches, ultimately enhancing patient outcomes.
